# A statistical image analysis framework for pore-free islands derived from heterogeneity distribution of nuclear pore complexes

**DOI:** 10.1038/s41598-017-16386-2

**Published:** 2017-11-24

**Authors:** Yasuhiro Mimura, Satoko Takemoto, Taro Tachibana, Yutaka Ogawa, Masaomi Nishimura, Hideo Yokota, Naoko Imamoto

**Affiliations:** 1 Cellular Dynamics Laboratory, RIKEN, Saitama, Japan; 2 Image Processing Research Team, RIKEN Centre for Advanced Photonics, Saitama, Japan; 3 Department of Bioengineering, Graduate School of Engineering, Osaka City University, Osaka, Japan

## Abstract

Nuclear pore complexes (NPCs) maintain cellular homeostasis by mediating nucleocytoplasmic transport. Although cyclin-dependent kinases (CDKs) regulate NPC assembly in interphase, the location of NPC assembly on the nuclear envelope is not clear. CDKs also regulate the disappearance of pore-free islands, which are nuclear envelope subdomains; this subdomain gradually disappears with increase in homogeneity of the NPC in response to CDK activity. However, a causal relationship between pore-free islands and NPC assembly remains unclear. Here, we elucidated mechanisms underlying NPC assembly from a new perspective by focusing on pore-free islands. We proposed a novel framework for image-based analysis to automatically determine the detailed ‘landscape’ of pore-free islands from a large quantity of images, leading to the identification of NPC intermediates that appear in pore-free islands with increased frequency in response to CDK activity. Comparison of the spatial distribution between simulated and the observed NPC intermediates within pore-free islands showed that their distribution was spatially biased. These results suggested that the disappearance of pore-free islands is highly related to *de novo* NPC assembly and indicated the existence of specific regulatory mechanisms for the spatial arrangement of NPC assembly on nuclear envelopes.

## Introduction

The eukaryotic nuclear envelope (NE) is composed of double lipid bilayers termed the outer (ONM) and inner (INM) nuclear membranes, which face the cytoplasmic and nucleoplasmic sides, respectively. The NE is supported structurally by the nuclear lamina, which consists of the A-type and B-type lamins, and transmembrane domain-containing proteins termed INM proteins. Nuclear pore complexes (NPCs), the gateways for all molecules that enter and exit the nucleus, are embedded in the NE^1^. Thus, an understanding of their function and the regulatory mechanisms of their assembly is a prerequisite for investigation of diverse cellular processes.

The NE in human cancer cell lines (HeLa S3 and U2OS) and human normal diploid fibroblasts (IMR90) contains two types of subdomains readily distinguished by their structural differences^2^: one is a pore-rich region enriched in B-type lamin and the INM protein lamin B receptor (LBR), whereas the other is a pore-free island enriched in A-type lamin and the INM protein emerin. The most predominant structural difference between these NE subdomains constitutes the exclusion of NPCs from pore-free islands^2^.

Pore-free islands, which are surrounded by pore-rich regions, are formed on the surfaces of the NE in early G1 phase cells^2^. Pore-free islands have the largest area in early G1 phase, which gradually disperse with cell cycle progression. Consequently, most pore-free islands disappear, and the NE constituents are evenly distributed prior to mitosis^2^. During the disappearance of pore-free islands, a cyclin-dependent kinase (CDK) regulates the transition of NE constituents in pore-rich regions but not in the pore-free islands^3^.

The NPC, which is assembled from multicopies of approximately 30 distinct proteins called nucleoporins (Nups), has an eight-fold rotationally symmetrical structure and accommodates the nucleo-cytoplasmic transport pathway for macromolecules^4^. Many Nups are assembled into subcomplexes, which are the building blocks of the NPC. The scaffold structure of the NPC is composed of three ring-like structures; with the cytoplasmic and the nucleoplasmic rings configured by the NUP107–160 subcomplexes^5^, and the spoke ring (also called the inner ring) configured by the NUP93–205 subcomplexes^6^. The NUP62 subcomplex forms the central channel on the spoke ring^7^ and other Nups comprise the cytoplasmic fibril^8^ and the nuclear basket^9^ on the cytoplasmic and the nucloplasmic rings, respectively^10,11^. Once the NPCs are embedded into the NE, their relative positions in the NE are maintained until subsequent NE breakdown during mitosis^12–14^.

Mammalian cells assemble NPCs twice throughout the cell cycle—in mitosis and interphase. Although equivalent numbers of structurally and functionally identical NPCs appear to be assembled in both phases^3,15^, each assembly process differs in certain aspects, such as Nups recruitment order, enzymatic activity requirement, and maturation time. In mitosis, the NPCs are re-assembled concomitant with NE re-formation on the surface of daughter chromosomes. In this context, NPC assembly is initiated by recruitment of the NUP107–160 subcomplex to the surface of daughter chromosomes via embryonic large molecule derived from yolk sac (ELYS)/MEL-28, one of its components with nucleosome-binding ability^16–18^. The transmembrane Nup POM121 and sub-stoichiometric amounts of the nuclear basket components, NUP153 and NUP50, are then recruited to the site, where NUP107–160 subcomplexes are seeded^19^. Subsequently, other Nups and their subcomplexes are recruited to this site^20,21^ and fully competent NPCs are assembled. In contrast, in interphase, the INM must be fused with the ONM to embed *de novo* NPCs in the closed NE. POM121, which appears to be involved in this NE fusion process^22^, is recruited to the site of NPC assembly, followed by the recruitment of NUP107–160 and NUP93–205 subcomplexes^14,23^ and, subsequently, other peripheral Nups and their subcomplexes^24^. Thus, certain mechanistic differences between interphase and mitotic NPC assembly likely exist. For example, CDKs, particularly CDK1 and CDK2, determine NPC assembly during interphase but not in mitosis^3^. In addition, compelling evidence suggests that the initial stage of interphase NPC assembly requires the nuclear transport function of matured NPCs^23,25–28^. The required times for NPC maturation during mitosis and interphase differ; NPC assembly in mitosis is completed within approximately 10–20 minutes^19^, whereas after mitosis, the number of NPCs doubles over interphase and a single NPC maturation requires 40–50 minutes^14,24^. This disparity is considered to reflect the differences of each assembly process.

Previously, we reported that the perturbation in NPC assembly by CDK inhibition resulted in the maintenance of pore-free islands until late G2 phase, suggesting that NPC assembly causes the disappearance of pore-free islands^3^. Experimental verification of this hypothesis by investigating pore-free islands provides new insights on NPC assembly. However, currently, a biological approach for clarifying the causal relationship between these two events remains to be established. Image analysis, which assists in better understanding of biological processes by measuring the shape, distribution, and dynamics of biological targets from images, is a powerful tool for complementing such biological approaches. Using image analysis, the internal and external features of pore-free islands were analysed to elucidate a ‘landscape’ for decoding the underlying mechanisms related to the disappearance of pore-free islands and NPC assembly. To describe the external features, we attempted to identify the boundaries between pore-rich regions and pore-free islands from images, which lead to a numerical translation of the size of pore-free islands. Unfortunately, these subdomains were distinguished only by differences in the mature NPC density, and thus no obvious structural boundary exists between them. This is not surprising, as the morphology of each pore-free island is extremely variable. In fact, many image-processing methods such as edge detection^29^, deformable models^30^, or thresholding^31^ are unable to recognise pore-free islands because of their diversity. An example of the internal features of pore-free islands is the numerical information related to internal focal structures, such as number and position, one of the crucial factors for understanding the underlying mechanisms of pore-free island disappearance. Unfortunately, the internal focal structures were tightly packed and buried within the image noise.

In this study, we propose a novel framework for image-based analysis to determine automatically the ‘landscape’ of pore-free islands from a large number of images. Information measured automatically using our framework, including the internal and the external spatial features of pore-free islands in the presence of active or inactive CDK, allowed us to clarify the relationship between the disappearance of pore-free islands and *de novo* NPC assembly. Our method was able to overcome the aforementioned obstacles in image analysis and automatically recognise the exact spatial position of elusive pore-free islands and their internal NPC intermediates from noisy immunofluorescence images. Three numerical factors were derived from our framework: pore-free island size, number of NPC intermediates, and their distribution. Based on these factors, we assessed the appearance frequency of NPC intermediates containing the Nups essential for NPC assembly and maintenance of pore-free islands and their impact on CDK inhibition. In addition, we compared the spatial distribution between simulated and observed NPC intermediates within the pore-free islands to detect potential spatial bias. The results of these analyses will provide information related to the relationship between pore-free island disappearance, *de novo* NPC assembly, and the existence of regulatory mechanisms for the spatial arrangement of NPC assemblies on interphase nuclear envelopes.

## Results

### NUP93, NUP107, and POM121 form focal structures in pore-free islands

**Figure 1:**
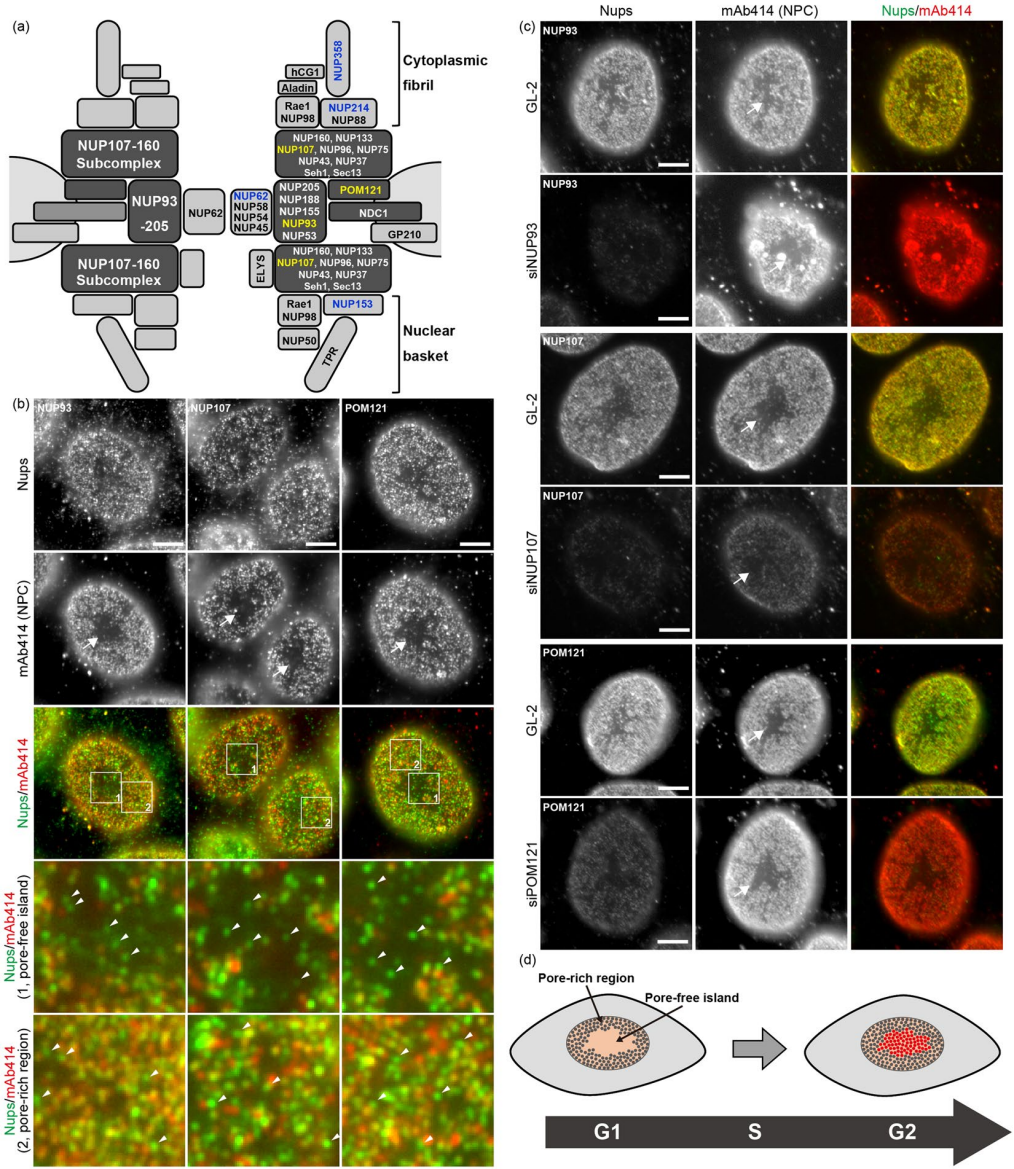
NUP93, NUP107, and POM121 can potentially form NPC intermediates within pore-free islands. (**a**) Schematic representation of the positions of Nups (right half) and Nups subcomplexes (left half) in NPC. Dark grey boxes show Nups subcomplexes involved in interphase NPC assembly. Nups shown in blue are recognised by the mAb414 antibody, and Nups shown in yellow were tested in this study. (**b**) HeLa S3 cells were co-immunostained with antibodies against NUP93, NUP107, or POM121 as well as mAb414 (NPC). Rectangles 1 and 2 in the merged images indicate the enlarged positions 1 (pore-free island) and 2 (pore-rich region), respectively. Arrows and arrowheads indicate the position of pore-free islands (mAb414-free regions) and mAb414-independent Nup foci, respectively. Scale bar, 5 μm. (**c**) HeLa S3 cells were transfected with control siRNA (GL-2) or siRNA for Nups (NUP93, NUP107, or POM121), and cultured for 72 h. All images show the bottom surface of the NE. Arrows indicate the positions of pore-free islands. Scale bar, 5 μm. (**d**) Schematic illustration of the hypothesis regarding the disappearance of pore-free islands with cell cycle progression. Grey and red dots indicate old and newly-generated NPCs, respectively. In our hypothesis, pore-free islands disappeared through the establishment of *de novo* NPCs.

Co-immunostaining with mAb414 antibody and antibodies against individual Nups including NUP93, NUP107, or POM121 was performed to survey the landscape of pore-free islands in the NE bottom surface. These Nups are critical for the assembly and maintenance of NPCs (Fig. 1a). The mAb414 antibody, which recognises FG-repeat-containing Nups, including NUP358, NUP214, NUP153, and NUP62^32^, is widely used to detect structurally and functionally matured NPCs. The distribution of mAb414 foci in the NE exhibits good correspondence with the arrangement of pore-rich regions and pore-free islands^2^, with mAb414-rich and mAb414-free regions corresponding to the pore-rich regions and pore-free islands, respectively (Fig. 1b; arrows in mAb414 images). Notably, we observed that components of the scaffold subcomplexes, NUP107 or NUP93, formed the respective focal structures in pore-free islands (Fig. 1b; arrows in enlarged pore-free island images). Similarly, POM121 formed focal structures in pore-free islands as well. We also observed these focal structures in pore-rich regions (Fig. 1b; arrows in enlarged pore-rich region images). All foci shown in Fig. 1b were depleted by transfection with siRNA targeting the respective Nups, confirming that the foci recognised by antibodies for NUP93, NUP107, or POM121 in pore-free islands and pore-rich regions contained the respective Nups (Fig. 1c).

POM121 is required for interphase NPC assembly and is recruited to the NPC assembly site prior to the recruitment of the NUP107–160 subcomplex^14,23,27^. All the Nups recognised by the mAb414 antibody are incorporated into the NPC depending on the scaffold subcomplexes; the incorporation of NUP358, NUP214, and NUP153 depends on the NUP107–160 subcomplex^10,33,34^ and that of NUP62 depends on the NUP93–205 subcomplex^7,10^. Our observations suggested that the foci detected only by anti-NUP93, NUP107, or POM121 antibodies indicated NPC intermediates in the middle of NPC formation. Investigating this possibility would reveal whether interphase NPC assembly caused the disappearance of pore-free islands (Fig. 1d).

### Quantitative image analysis framework for understanding pore-free islands

**Figure 2:**
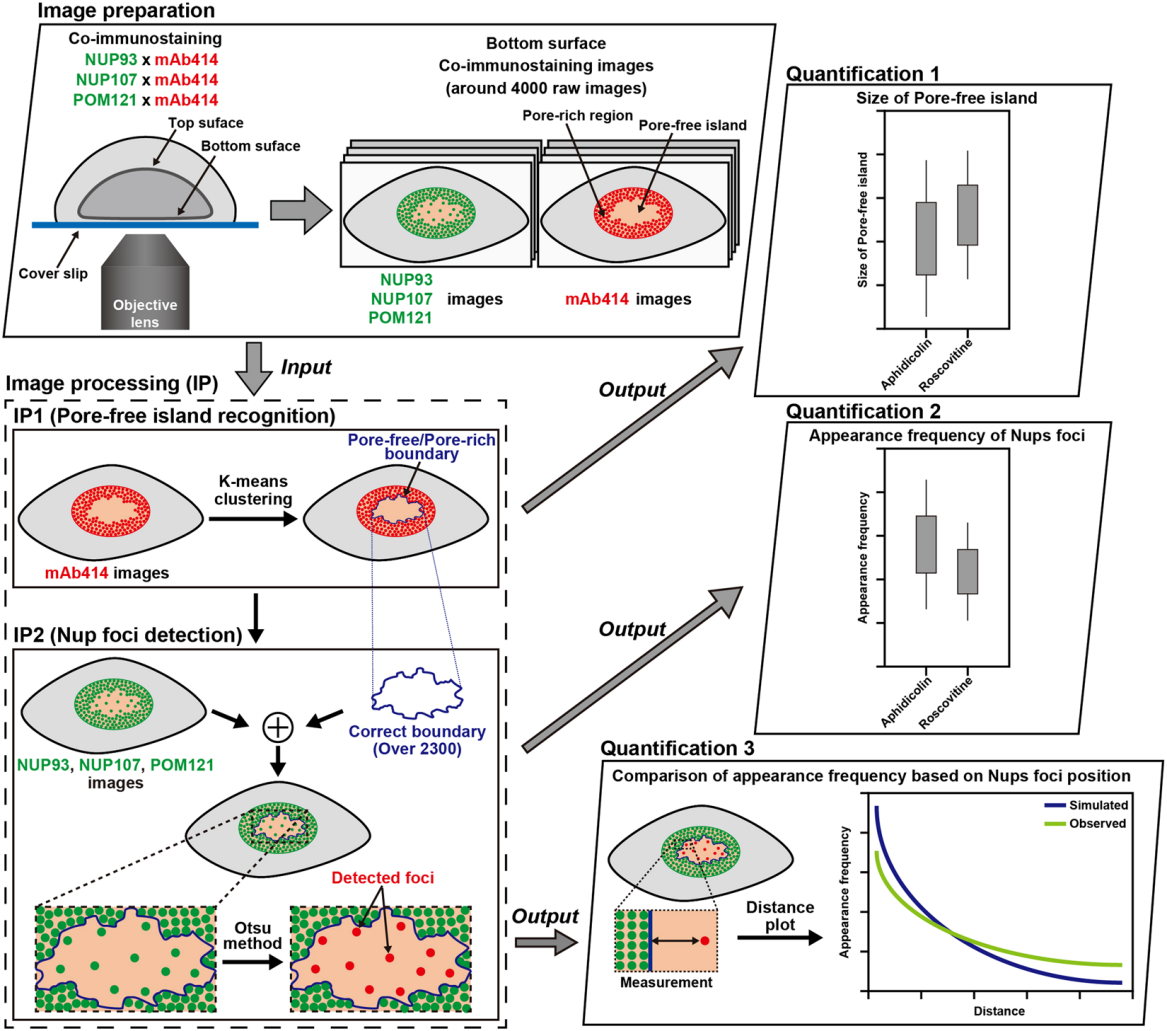
Image analysis framework for understanding pore-free islands. Our framework was constructed using the following three categories: image preparation, image processing (IP), and quantification. Image preparation shows the acquisition of three sets of co-immunostained images that were collected at the bottom-surface of NE. After manually specifying the nuclear regions in the mAb414-images using our image processing software (http://logistics.riken.jp/vcat/vcat/en), IP1 was next performed to recognise the area of pore-free islands in each specified nuclear region. The recognition results were subjected to statistical analysis of the pore-free island size with respect to inhibitor type, the time of inhibitor treatment, and primary antibodies (Quantification 1). Subsequently, the recognised areas of pore-free islands were used as the initial regions for automatic detection of Nups foci in the NUP93, NUP107, and POM121 images. Automatic Nups foci detection was performed using the Otsu method, focusing on the geometric feature (see Materials and Methods). According to the measurements of the detected Nups foci in each pore-free island, quantified data regarding their appearance frequency were obtained (Quantification 2). In addition, we investigated the position of Nups foci in the pore-free islands to determine whether their distribution was spatially biased by comparing the observed and simulated data (Quantification 3).

We next assessed the effect of CDK activity, which regulates *de novo* NPC assembly in interphase, on the Nups foci in pore-free islands using a large number of immunofluorescence images. In this study, the cells were treated with aphidicolin, an inhibitor of DNA polymerase that does not affect pore-free islands (control reagent)^3^, or roscovitine, a CDK inhibitor, for 6 or 10 h, followed by co-immunostaining with an mAb414 antibody and antibodies against NUP93, NUP107, or POM121. In our experimental setup, approximately 4,000 co-immunostaining images of the bottom surface of the NE were collected (Fig. 2, image preparation). Our image analysis framework was applied to all the collected co-immunostaining images to statistically evaluate the effect of CDK activity on the Nups foci within pore-free islands (Fig. 2, Image processing). To establish a quantitative analysis framework, we overcame two primary difficulties with respect to image processing: (1) recognition of pore-free islands in immunofluorescence images (Fig. 2, IP1), and (2) detection of internal Nups foci within the recognised pore-free islands (Fig. 2, IP2).

**Figure 3:**
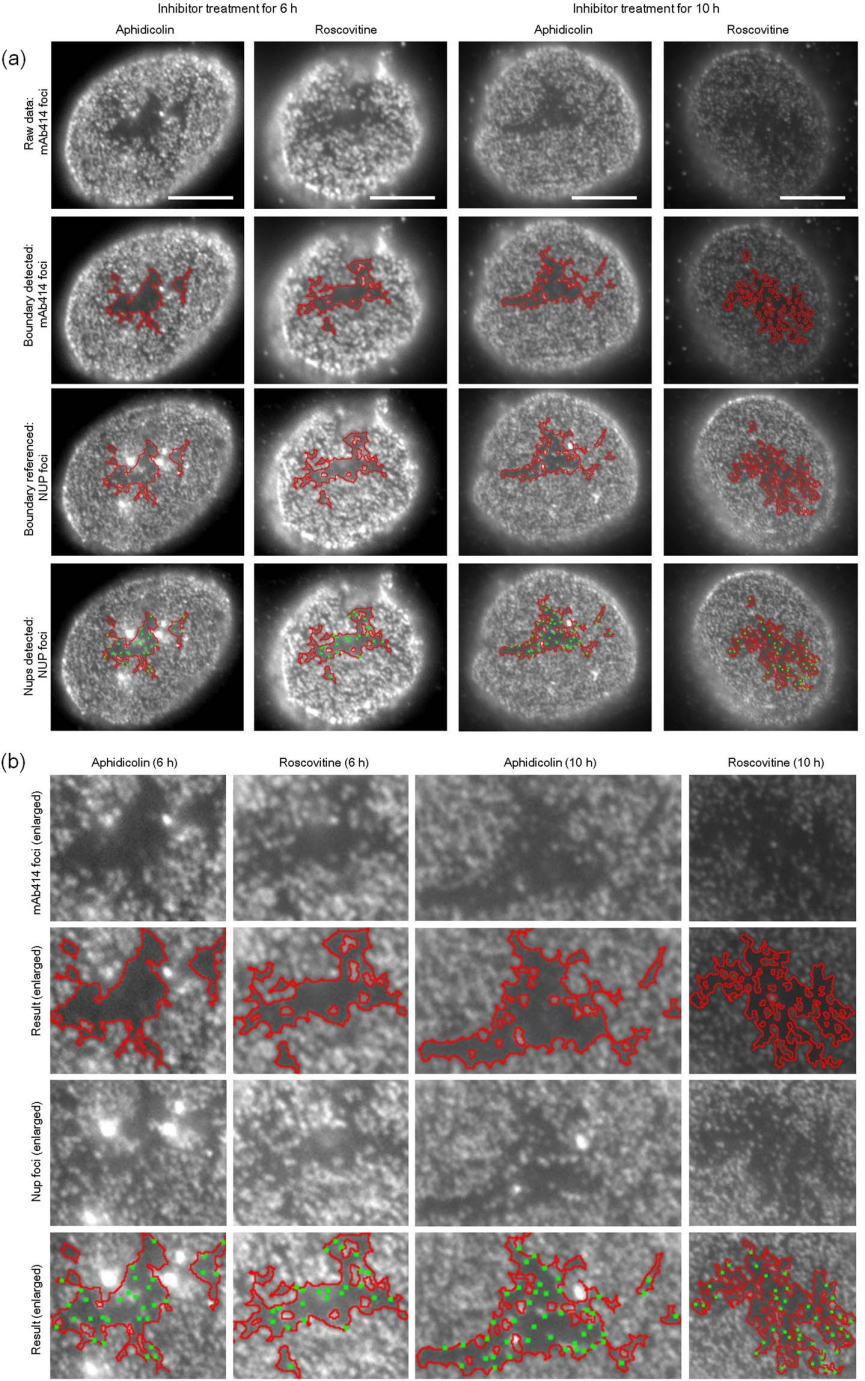
Results of pore-free island recognition and Nups foci detection. (**a**) The first row shows the mAb414-staining images with aphidicolin or roscovitine for 6 or 10 h; the second row shows the results of pore-free island recognition. The red lines indicate the boundary between the pore-rich region and the pore-free island. The third row presents POM121-staining images, showing the region with pore-free island recognised in the mAb414-staining images with red lines. The fourth row shows the automatic detection results of POM121 foci within each pore-free island. Representative POM121 foci are indicated by the green points. Scale bar, 5 μm. (**b**)The first and second rows show the enlarged image sets of the mAb414-staining images, and results pertaining to the recognition of pore-free islands are shown in Fig. 3a. The third and fourth rows show the enlarged image sets of Nup-foci images, and the results pertaining to the detection of POM121 foci are shown in Fig. 3a.

Regarding difficulty (1), we achieved automatic segmentation of pore-free islands by applying K-means clustering^35^ using the three types of image moment features extracted from the mAb414-staining images (Fig. 3a, second row). These features included mean, variance, and second order central image moment around their centroid within a 3 × 3 pixel area, which are related to brightness, contrast, and intensity spread of images, respectively. Our method performed pore-free island segmentation in an average of five minutes per cell, whereas one hour per cell is the average time required by the manual method; thus, we effected a greater than 10-fold improvement in time efficiency. In addition, to avoid losing objectivity, we devised ways of deciding the number of clusters when performing K-means (see Materials and Methods). To verify whether our method correctly recognised the area of each pore-free island in mAb414-staining images, the processing results were further confirmed by our visual judgment. Consequently, approximately 2,300 results that correctly recognised the area of pore-free islands were adopted (see Supplementary Table S1).

Regarding difficulty (2), we achieved automatic detection of Nup foci by applying the Otsu method^31^, a well-known binarization method, to the images immunostained for NUP93, NUP107, or POM121 (Fig. 3a, fourth row). Next, the area information of pore-free islands obtained from the above procedure was referenced against the Nups-counterstaining images as an initial region of foci detection (Fig. 3a, third row). In the Nups-counterstaining images, the same coordinate pixels as those of the pore-free islands in the mAb414-staining images, were subjected to binarization, which were then translated to mean curvature, a differential geometric feature^36,37^, to distinguish them from unnecessary pixels such as image noise (see Materials and Methods). The Nup foci detection results were also checked by visual observation, confirming the accuracy of our automatic detection results (Fig. 3b).

Finally, to statistically analyse the effects of CDK activity on pore-free islands, substantial quantities of image processing results obtained from the two image-processing procedures were translated into three factors: size of pore-free islands, appearance frequency of Nup foci, and their spatial position within the pore-free islands (Fig. 2, Quantification 1–3). Overall, our image analysis framework encompasses two types of automated image-processing methods and three types of quantification procedures, offering valuable information for understanding the events occurring adjacent to pore-free islands. To acquire data objectivity, our framework maximally reduced the number of arbitrary procedures requiring manual operations during image-processing. In addition, all the processing results were checked and uncorrected results were excluded from the statistical analysis. This confirms that our analysis, which uses three types of quantified values related to the landscape of pore-free islands, exhibits sufficiently high accuracy for generating confident results.

### Quantification 1: CDK activity is required for the disappearance of pore-free islands

**Figure 4:**
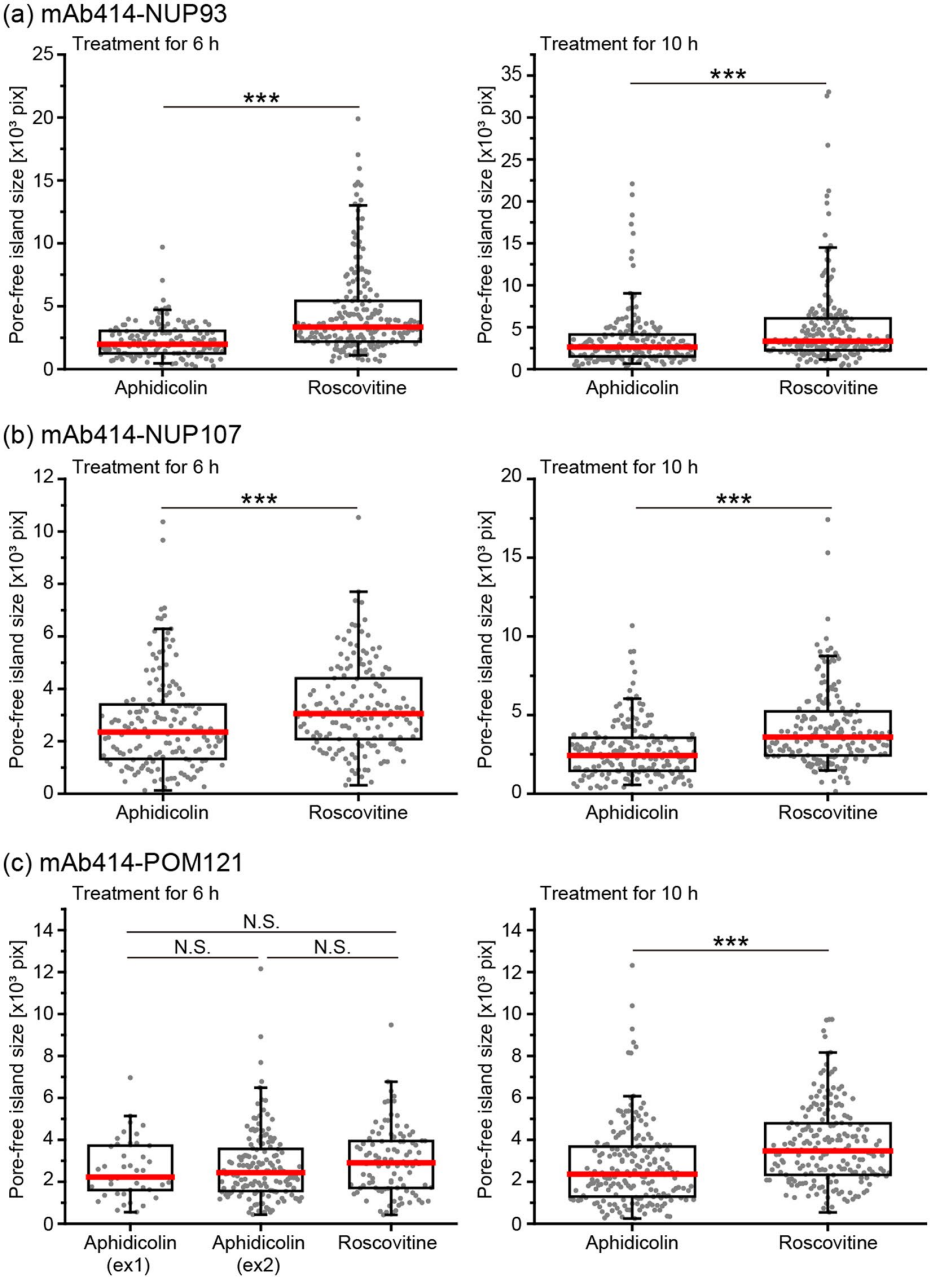
Size comparison of pore-free islands between cells treated with aphidicolin or roscovitine. Bee-swarm and box-whisker plots of the size of pore-free islands in cells treated with aphidicolin or roscovitine for 6 or 10 h, and co-stained with NUP93 (**a**), NUP107 (**b**), or POM121 (**c**) and mAb414 antibodies. The box shows interquartile range (25% and 75%) of the data set and red bars show the median of the data set. Whiskers show 95% and 5% of the data set, respectively. The data were analysed using the Brunner-Munzel test; ***P < 0.001; N.S., not significant (P > 0.05).

We measured the pore-free island size that is correctly recognised by the developed evaluation strategies, and then compared the same between aphidicolin- and roscovitine-treated cells (Fig. 2, Quantification 1). The pore-free island size in the NUP93-mAb414-staining images acquired from the cells treated for 6 or 10 h with roscovitine was larger than those from cells treated for an equivalent time with aphidicolin (Fig. 4a). Furthermore, the pore-free island size measured from the images of NUP107-mAb414 and POM121-mAb414 staining also showed similar results, except that the POM121-mAb414-staining images were acquired from cells treated with aphidicolin or roscovitine for 6 h (Fig. 4b and c). To rule out the possibility that the size difference of the pore-free islands between aphidicolin- or roscovitine-treated cells was affected by the combination of secondary antibodies, we measured the pore-free island size in secondary antibody-swapped POM121-mAb414-staining images acquired from cells treated with aphidicolin or roscovitine for 6 h (Fig. S2a). The result obtained from these images was similar to the result shown in Fig. 4, suggesting there was no obvious effect of either combination of secondary antibodies in our image-processing framework. Additionally, the cells cultured for 18 h after the same synchronisation procedure as used for the cells shown in Fig. 4, showed smaller pore-free islands than those shown in Fig. 4 (see Table S1, mAb414–18h). Therefore, these results indicate that CDK activity is positively required for the disappearance of pore-free islands, which is consistent with the results of a previous report that used a different methodology^3^.

### Quantification 2: CDK inhibition decreases the appearance frequency of NUP93, NUP107, or POM121 foci in pore-free islands

**Figure 5:**
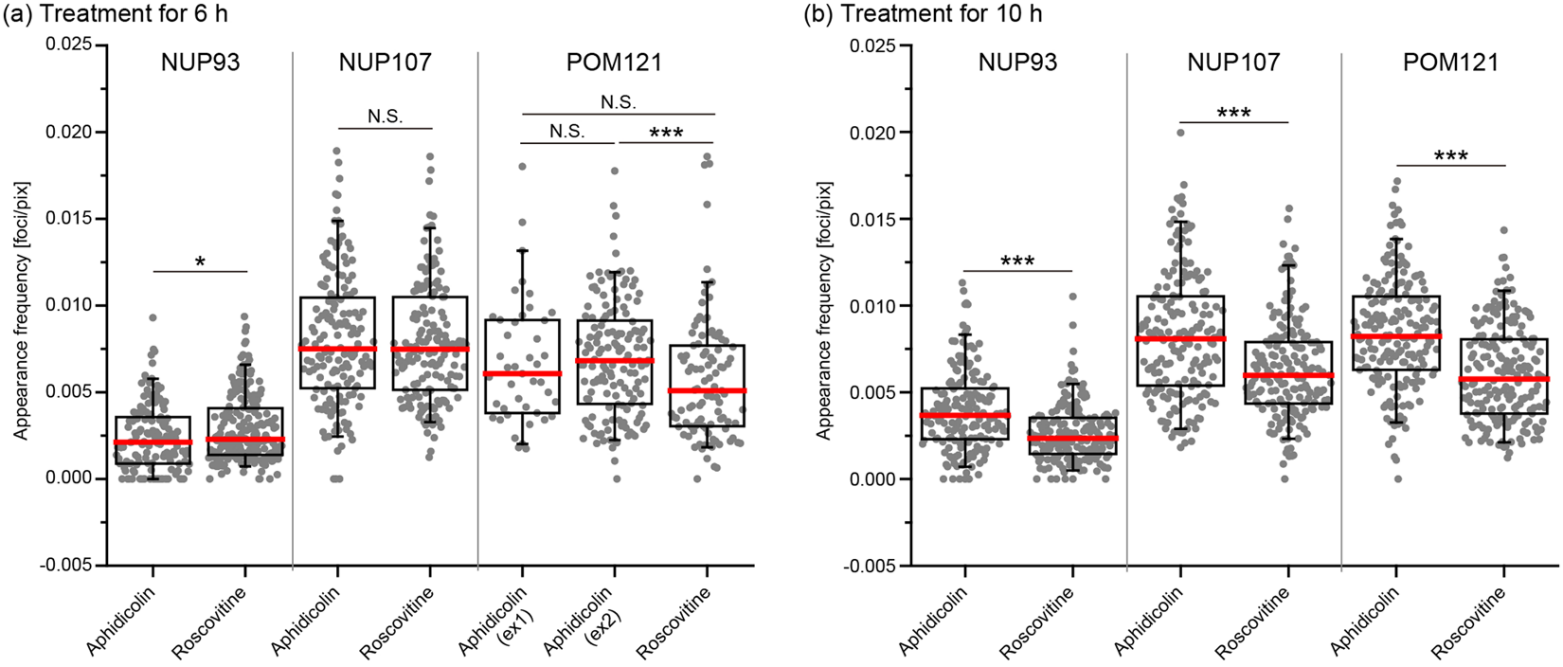
Appearance frequency of Nup foci in pore-free islands. Bee-swarm and box-whisker plots of the appearance frequency of NUP93-, NUP107-, or POM121-foci in pore-free islands in cells treated with aphidicolin or roscovitine for 6 h (**a**) or 10 h (**b**). The box shows interquartile range (25% and 75%) of the data set and red bars show the median of the data set. Whiskers show 95% and 5% of the data set, respectively. The data were analysed using the Brunner-Munzel test; *P < 0.05; ***P < 0.001; N.S., not significant (P > 0.05).

The appearance frequency of Nups foci in the automatically detected pore-free islands stained with anti-NUP93, NUP107, or POM121 antibodies was measured (Fig. 2, Quantification 2). Each appearance frequency was calculated as the detected number of Nups foci relative to the corresponding pore-free island size. Large differences were not detected in the frequency of any of the three foci types between cells treated with aphidicolin or roscovitine for 6 h (Fig. 5a). In addition, the frequency of POM121 foci acquired from the secondary antibody-swapped POM121-mAb414-staining images in cells treated with aphidicolin or roscovitine for 6 h also showed similar results (Fig. S2b). In contrast, the frequencies of cells treated with aphidicolin for 10 h were significantly higher than those following roscovitine treatment for 10 h (Fig. 5b). According to a previous report^3^, CDK activity is required for targeting the NUP107–160 subcomplex to the NPC assembly site, which promotes interphase NPC assembly. Thus, the increase in the appearance frequency of NUP107-foci in pore-free islands by CDK activity is possibly because of *de novo* NPC assembly, suggesting that the detected NUP107-foci contain a population of NPC intermediates. The appearance frequencies of NUP93- or POM121-foci were also similar to those of NUP107-foci. These results are consistent with the results of a recent report showing that interphase NPC assembly occurs actively in the core region, which is structurally similar to pore-free islands in late mitosis^24^. Thus, we concluded that interphase NPC assembly is positively required for the disappearance of pore-free islands.

### Quantification 3: NPC intermediates in pore-free islands localise away from pore-rich regions

**Figure 6:**
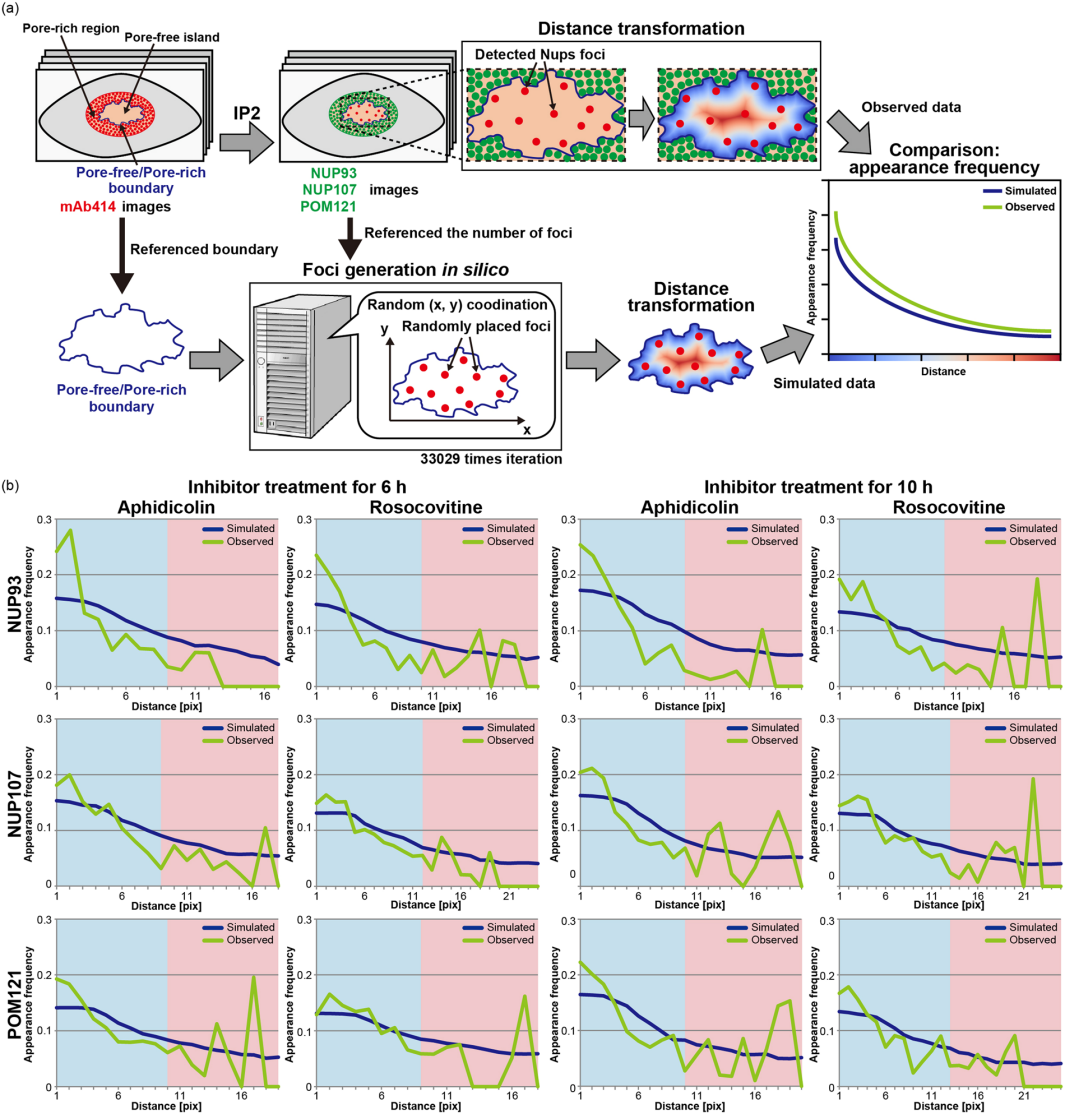
Comparison of appearance frequency between observed and simulated Nup foci. (**a**) Schematic illustration of the distance measurement from each Nup focus to the boundary between pore-free islands and pore-rich regions. The shortest distances of each Nup focus detected in Fig. 5 were measured by distance transformation and the appearance frequencies in each distance were calculated (observed data). The observed data were compared with the data simulated using actual locations of pore-free islands and the number of Nups foci detected in each pore-free island (simulated data). (**b**) Positions of Nup foci with respect to NUP93, NUP107, or POM121 in pore-free islands were quantified in cells treated with aphidicolin or roscovitine for 6 or 10 h using the experimental real data. The quantified appearance frequencies were averaged and normalised, and then plotted for each experimental condition. Green and blue lines show the observed and simulated data, respectively.

We investigated the spatial position of the Nup foci detected in Quantification 2 to explore the regulatory mechanism of the assembly of the NPC intermediates. This constitutes a novel and ingenious approach for studying NPC assembly. We first conducted a distance transform^38^ on each region of the recognised pore-free islands in the respective Nup-staining images. The distance transform generates a distance field represented by a scalar value, with the field specifying the minimum distance to a shape. In our examination, the distance field was used for measuring the minimum distance between the boundary of pore-free islands and internal foci. Each boundary was set between the pore-free island and the pore-rich region and defined as a starting point for the distance transform (Fig. 6a, distance transformation). All the detected Nups foci were counted in every distance and then the total number was translated into an appearance frequency by dividing it by the number of pixels in each distance to which it belongs. The calculated frequency of individual pore-free islands was averaged at each experimental setting and summarised as a normalised appearance frequency with a sum of 1.0 (Fig. 6b, observed).

We next determined whether the Nups foci in pore-free islands were distributed randomly by comparing the observed and simulated Nups foci (Fig. 6a). The simulated Nups foci were generated at all random positions within individual pore-free islands recognised in our observed results (used in Fig. 4). The coordinates of the simulated Nups were based on the Mersenne Twister^39^, a pseudo random number generator. The number of pore-free islands simulated was identical to that of the corresponding actually detected foci, which were counted in every distance field in the same manner as that used for deriving the observed results. These operations were repeated 33,029 times per single pore-free island (Fig. 6a), and the number of Nups foci was averaged at every distance field and translated into the appearance frequency using a procedure identical to that used for obtaining the observed results. All operations were performed and summarised for each pore-free island similar to that used for obtaining the experimental results (Fig. 6b, simulated).

To assess the spatial bias for the appearance frequency of Nups foci within pore-free islands, we divided the graph into two regions—proximal and distal— from the pore-free/pore-rich boundary (Fig. 6b, blue and red background). Then, we compared the distribution of the observed appearance frequencies of Nups foci with those of their simulated counterparts (hereafter the observed distribution and the simulated distribution, respectively) at each of the two regions under every experimental condition (also see Table S2). We were not able to detect any significant differences between the observed and simulated distributions at any experimental condition in the proximal regions. In contrast, the observed distributions at some experimental conditions were different from the corresponding simulated distributions in the distal regions, except for the distributions for three experimental conditions (NUP93-mAb414, roscovitine for 6 h; NUP107-mAb414, aphidicolin for 10 h; POM121-mAb414, aphidicolin for 10 h).

It is noteworthy that CDK inhibition had no significant effect on the distribution of NPC intermediates throughout pore-free islands. Our results indicate that the distribution of Nups foci within the pore-free islands was spatially biased compared to that of the simulated foci in the distal region. Such spatial bias might reflect the dynamics of disappearing pore-free islands mediated via interphase NPC assembly.

## Discussion

In this study, we investigated the landscape of pore-free islands to understand the causal relationship between two events regulated by CDK: disappearance of pore-free islands and interphase NPC assembly. For this purpose, we proposed an image analysis framework (Fig. 2) and succeeded in improving the analysis throughput of pore-free islands from multiple images. Our proposed framework was applied to cells with active or inactive CDK to extract three numerical factors regarding the landscape of pore-free islands: size, appearance frequency of Nups foci, and spatial position. These quantified factors clearly showed that interphase NPC assembly was positively required for the disappearance of pore-free islands. Therefore, a pore-free island is a useful region for the investigation of the details of NPC assembly in interphase, including the assembly process and the spatial regulation of NPCs.

Interphase NPC assembly is a highly ordered process; POM121 is first recruited to the NPC assembly site, followed by the scaffold Nups, NUP107, and NUP93, and finally the peripheral Nups such as NUP358^10,14,24^. In this process, CDK is required in the step prior to the recruitment of the NUP107–160 subcomplex to the NPC assembly site^3^. Here, we revealed that CDK activity increases not only the appearance frequency of the NUP107 foci but also those of the POM121 and NUP93 foci (Fig. 5b), suggesting that CDK functions at the step prior to the recruitment of POM121 into the NPC assembly site. In addition, the appearance frequency of NUP93 foci was clearly lower than that of the POM121 and NUP107 foci under both CDK active and repressive conditions (Fig. 5), suggesting that different appearance frequencies might reflect the order of interphase NPC assembly. These results led to a hypothesis regarding the process of interphase NPC assembly, wherein CDK activity promotes the recruitment of POM121 to the NPC assembly site, followed by recruitment of the NUP107–160 subcomplex. Subsequently, the NUP93–205 subcomplex is incorporated into this NPC intermediate, followed by peripheral Nups, as recognised by an mAb414 antibody. To verify this hypothesis, the NPC intermediates that were detected in the pore-free islands should be further characterised using the tested antibodies. To this end, our proposed framework must be improved for future studies to assess all the other co-localising Nups residing in the NPC intermediates.

Our results showed that the appearance frequency of NPC intermediates is spatially biased in pore-free islands compared to that observed with the simulated results (Fig. 6b and Table S2). Thus, the position of the NPC assembly site might be spatially regulated in pore-free islands, which may be inferred from the interaction between Nups and the nuclear lamina as lamins regulate NPC positions in the *Drosophila* brain^40^. Among the Nups, two components that are recruited at early steps of interphase NPC assembly, NUP153^28^ and POM121^19,23,27^, are good candidates for this regulation. In particular, NUP153 can biochemically interact with both A-type and B-type lamins^41,42^, whereas POM121 can biochemically interact with LBR^27^ and is phosphorylated by CDK in interphase (data not shown). Future work with our proposed framework should therefore include verification of the roles of these two Nups as well as screening of other candidates during the spatial regulation of the NPC assembly site.

The structure and composition of pore-free islands is important to deduce its physiological significance. Structural features of pore-free islands such as the lack of NPC, enrichment of A-type lamin, and lower amount of B-type lamin and LBR^2,3^, resembles those of nuclear blebs in fibroblasts derived from patients with Hutchinson-Gilford progeria syndrome (HGPS) carrying the C428T (p.S143F) mutation in *LMNA* ^43^. Bleb formation due to mutant lamin A expression is involved in structural alteration of chromatin and transcriptional activation. These observations raised the possibility that the formation of pore-free islands represents phenotypes similar to those observed in the HGPS fibroblast. Therefore, to understand the physiological significance of pore-free islands, it is important to analyse the chromatin structure, including histone modifications, and transcriptional activity underneath this nuclear subdomain.

In our framework, information regarding the landscape of pore-free islands was extracted from multiple fixed cell images rather than time-lapse imaging. Although time-lapse imaging of live cells is widely used to understand diverse intracellular dynamics at a single cell level (e.g., molecular kinetics and morphological alterations of organelles), a trade-off exists between image quality in each frame and phototoxicity to the cells upon exposure to illumination. In contrast, fixed cell-based techniques—such as immunostaining—allow relatively straightforward acquisition of high quality images without the intervention of phototoxicity, although considerable time and effort is required to obtain information equivalent to that obtained from time-lapse imaging. Our proposed framework contains some automated image-processing methods, which allows high-throughput analysis and facilitates acquisition of the desired information regarding pore-free islands. These aspects enabled us to best utilise the advantages of fixed cell-based techniques. Specifically, prompt analysis using multiple images readily led to statistical estimation of subject dynamics, thus compensating for the shortcomings of fixed cell-based techniques.

It is noteworthy that our image-processing methods, which constitute the most important components of our proposed framework, were able to recognise pore-free islands and detect internal foci automatically, even though the former were barely defined as an explicit area and the latter were buried within image noise. A large number of images, including undesirable images with intensity saturation and blurring, were subjected to our image-analysis framework to increase the number of images adopted for analysis. Although this reduced the accuracy of the method, reliability of the analysis results was supported by numerous images. We consider that the principal reasons for the rejection of results were the following three image-associated problems: intensity saturation, nuclei edge-blurring, and minute pore-free islands (Fig. S3d).

Our image-processing methods may be applied to any other subjects regarding cellular image processing. In particular, our recognition method for pore-free islands would be useful for subjects that exhibit many bright points with differing densities or that have to be distinguished into specific clusters. Alternatively, for more generalised techniques, our method should be further improved to avoid image noise-related effects. We consider that one of the most influential contributors to the accuracy of our method constitutes the selection of image features. In our recognition procedure of pore-free islands, we used three types of image moment features and then classified them into two clusters (i.e., pore-free islands and others) using K-means. To further improve the accuracy, more appropriate feature representation and its classification (or clustering), which can distinguish the area of interest from others while being minimally affected by noise should be defined, although it represents a challenging proposition. In future studies, this may be solved by the use of a sophisticated learning technique such as convolutional neural networks (CNN)^44^ that can automatically provide useful representations directly from raw images. A type of CNN has already been used for cell image processing^45^. However, although effectively training a CNN is difficult when the numbers of training images are insufficient, we have already obtained the adequate classified images in this study, and therefore, we are certain that in combination with CNN, our framework may acquire enhanced detection power and accuracy.

In summary, our proposed framework demonstrates that the disappearance of pore-free island is directed through interphase NPC assembly. This conclusion is supported by our observation that the size of pore-free islands (Fig. 4) and the appearance frequency of Nups foci in pore-free islands (Fig. 5) decreased following CDK inhibition, which indicates the existence of NPC intermediates in the midst of assembly in pore-free islands. Furthermore, comparison of the positions between the experimental observations and randomly simulated NPC intermediates within pore-free islands revealed their spatial bias in distribution (Fig. 6b). This result is indicative of specific regulatory mechanisms for the spatial arrangement of NPC assembly on interphase nuclear envelope. Finally, the novel statistical image analysis framework used herein may serve as a basis for developing highly accurate methods for general cellular image segmentation or detection.

## Materials and Methods

### Cell culture and sample preparation

HeLa S3 and U2OS cells were obtained and cultured as described previously^2^. Mouse myeloma cell line Sp2/0-Ag14 was cultured in RPMI-1640 medium (Wako, Osaka, Japan) containing 10% fetal bovine serum (FBS) (GE Healthcare, Little Chalfont, UK) at 37 °C and 5% CO_2_ as described previously^46^. To synchronise cells at the G1/S boundary, HeLa S3 cells were cultured in medium containing 2 mM thymidine (T1895, Sigma-Aldrich, St Louis, MO, USA) for 16 h followed by culture in normal medium for 8 h. After an additional thymidine treatment, the cells were incubated in medium containing 100 ng/ml nocodazole (Sigma) for 4 h to effect mitotic arrest. Arrested cells were collected by shaking off and washing thrice with PBS, and then re-plated into fresh medium. After incubation for 3 h, synchronised cells were cultured in medium containing 40 μM roscovitine (R7772, Sigma)^47^ or 5 μg/ml aphidicolin (A0781, Sigma)^48^ for 6 or 10 h, followed by immunostaining. Aphidicolin was used as a drug-treatment control for roscovitine, because it could arrest the cell cycle at the G1/S boundary via DNA polymerase α/δ inhibition without affecting nuclear growth, NPC formation, or pore-free island dynamics in the interphase^3,14,48^.

### Plasmid construction

The 5′-fragment of *NUP93* (1–1227 bp) was amplified by PCR using the *NUP93* cDNA (accession number: AK292262) as a template and the following primers: NUP93-N forward, 5′-AAA TTT GGA TCC ATG GAT ACT GAG GGG (BamHI site underlined) and NUP93-N reverse, 5′-AAA TTT GCG GCC GCT CTT TGT CCG CCA CTT C (NotI site underlined). After amplification, the 5′-fragment of *NUP93* was digested by BamHI and NotI and then subcloned into pGEX6p-1 (GE Healthcare).

### Protein purification

BL-21 (DE3) cells were transformed with pGEX6P-1 harbouring the 5′-fragment of *NUP93* (1–1227 bp). All colonies obtained were cultured in LB medium containing 50 μg/ml ampicillin (Nacalai Tesque, Kyoto, Japan) for 2 h at 37 °C and then further cultured in LB medium containing 1 mM isopropyl β-D-1-thiogalactopyranoside (IPTG) (Nacalai Tesque) for 2 h at 37 °C. Cells were harvested and re-suspended in lysis buffer (50 mM Tris-HCl, pH7.4, 500 mM NaCl, 1% TritonX-100, 1 mM EDTA, 1 mM DTT, 0.3 mM PMSF), followed by sonication. Note that the recombinant N-terminal NUP93 (1–409 amino acids) formed inclusion bodies, which was washed seven times with lysis buffer and used as an antigen for generating the anti-NUP93 antibody.

### Generation of a rat monoclonal anti-NUP93 antibody

All animal procedures in this study were approved by the Institutional Animal Care and Use Committee (permission number: S0036) and performed according to Osaka City University Animal Experimentation Regulations. A rat monoclonal antibody that specifically recognised NUP93 was generated based on the rat medial iliac lymph node method^49^. The rear footpads of a 10-week-old female WKY rat (Japan SLC, Shizuoka, Japan) were injected with 100 ml emulsions containing the GST-fused N-terminal NUP93 and Freund’s complete adjuvant. At 17 days after the first immunisation, an additional immunisation with N-terminal NUP93 minus the adjuvant was performed via the tail base of the rat. At 4 days after the additional immunisation, cells from the iliac lymph nodes of the immunised rat, euthanatized by administering an effective amount of anaesthetic, were fused with Sp2/0-Ag14 cells at a ratio of 5:1 in 50% polyethylene glycerol. The resulting hybridoma cells were plated onto 96-well plates and cultured in hypoxanthine-aminopterin-thymidine medium (HAT) selection medium. The NUP93-specific antibody was screened by enzyme-linked immunosorbent assay (ELISA), western blotting, and immunostaining using hybridoma supernatants. Finally, a hybridoma clone producing a monoclonal antibody, termed 2G1A9, was selected.

### Antibodies

The following primary and secondary antibodies were used: rat anti-POM121^50^, 1/1000; rabbit anti-NUP107, kindly provided by Dr. Doye^51^, 1/1000; rat anti-NUP93 (2G1A9) generated in this study, 1/500; mAb414 (MMS-120P, Covance, Princeton, NJ, USA), 1/3000; donkey anti-rabbit Alexa 488 (A-21206, Molecular Probes, Eugene, OR, USA), goat anti-rat Alexa 488 (A-11006, Molecular Probes), 1/800; goat anti-rat Alexa 594 (A-11007, Molecular Probes), 1/800; goat anti-mouse Alexa 488 (A-11001, Molecular Probes), 1/800; and goat anti-mouse Alexa 594 (A11032, Molecular Probes), 1/800.

### Immunostaining

Cells were fixed with 2% paraformaldehyde (PFA)/PBS for 10 min at 37 °C and perforated using 0.5% TritonX-100/PBS for 5 min. The cells were blocked with 5% normal goat serum (NGS, Chemicon, Billerica, MA, USA) in PBS for 1 h, incubated with the primary antibody diluted with 1% NGS/PBS for 2 h, washed thrice with PBS, then further incubated with the secondary antibody diluted in 1% NGS/PBS for 2 h. After washing thrice with PBS, the cells were stained with 4′,6-diamidino-2-phenylindole (DAPI) (Roche, Madison, WI, USA) and then mounted in PPDI [80% glycerol/PBS, 1 mg/ml paraphenylenediamine (11873580001, Roche)]. For immunostaining with the anti-NUP93 antibody, cells were fixed with 2% PFA/PBS for 5 min and then stained as described above.

Immunostaining images were acquired using a DeltaVision microscope (IX-71 DeltaVision CORE system; Olympus and Applied Precision, Tokyo, Japan) with a 100×/1.35 Plan Apo objective lens (Olympus) and a Cool Snap HQ2 CCD camera (Photometrics, Tuscon, AZ, USA) as a series of five images along the z-axis with 0.1- or 0.2-μm intervals. The lateral chromatic aberration in our microscopy system was evaluated using three-colour beads with 0.1-μm diameter (TetraSpeck^TM^ microspheres, Molecular Probes) and was determined to be negligible. The images focused on the NE bottom surface were selected manually from this series of five images and used for our framework.

### siRNA transfection

siRNA transfection was performed using RNAiMAX (Life Technologies, Gaithersburg, MD, USA) according to manufacturer’s instructions. siNUP93 (HSS114514, Invitrogen, Carlsbad, CA, USA), siPOM121^50^ (oligo-2, Integrated DNA Technologies, Coralville, IA, USA), and siNUP107^33^ (Integrated DNA Technologies) were used.

### Decision rule of the number of clusters in K-means for pore-free island recognition

To recognise pore-free islands (Fig. 2, IP1), image segmentation was performed using K-means clustering. Generally, the number of clusters *k*, which is fixed a priori, affects the clustering results significantly, and usually leads to a lack of flexibility in clustering. To solve this issue, we proposed an automatic decision rule for establishing *k*, inspired by the elbow method that finds the appropriate *k* ^52^. We computed K-means for different *k* (from 2 to 20). Then, the class having the region with the lowest mean intensity was regarded as a single class, including the pore-free islands (Fig. S3a). Subsequently, the most adequate *k* was selected by applying the criterion as follows. We calculated each ‘within-class’ variation (WCV) in terms of the three types of image features (mentioned in the Results section) of the class to which the pore-free island belonged with respect to each clustering result from *k* = 2 to 20. Next, we plotted a line chart of the total WCV calculated from the linear combinations of each normalized WCV. The original elbow method seeks the point where the line chart starts showing a diminishing gradient when *k* is increased. In our method, we regarded the point where the sign of the difference between the subsequent clusters (Diff_*WCV*_ = WCV_*k*+1_ − WCV_*k*_) changes (from minus to plus) as a selected cluster for pore-free island recognition. According to this decision rule for the *k*, all the pore-free islands were recognised automatically within multiple images.

All the recognition results were checked using visual judgement and 2,300 results were collected from 4,000 original images (Supplementary Table S1). To negate the possibility that our recognition may be sensitive to certain specific experimental settings, we conducted significance testing based on our experimental settings, including inhibitor treatment times (6 vs. 10 h), kinds of inhibitor (aphidicolin vs. roscovitine), and primary antibodies in combination with mAb414 (NUP93 vs. NUP107 vs. POM121) (Fig. S1). We found no significant differences, confirming that our recognition of pore-free islands was robust, at least within the experimental settings of this study.

### Nups foci detection based on differential geometric feature

The Nups foci within pore-free islands were automatically detected in the immunostaining images (Fig. 2 IP2). First, we referenced the recognised pore-free islands as the initial region of Nups detection. Next, we translated the images into mean curvature, a differential geometric feature^36,37^. Finally, we applied the Otsu method^31^ to the translated images to detect only the regions of existing Nups foci. The detected Nups were also checked visually.

Pixel intensities were not suitable for distinguishing Nups from other features as they were buried in the image noise. Conversely, we found that a curvature-based image feature was able to represent distinctive patterns of Nups. The mean of the principal curvatures was used for representing image features. Figure S4a shows a conceptual diagram of the curvature and Fig. S4b shows an example of the calculated mean curvature regulated to eight bits. To validate the precision of our proposed method, we compared the NPC density of U2OS cells obtained from the results generated using our proposed method with that obtained from the manually-detected results (Fig. S4c). The calculated NPC density from the result obtained using our method (2.56 NPCs/μm^2^) was comparable to that obtained using the manually-detected result (2.60 NPCs/μm^2^) (Fig. S4d).

### Download of the binary files regarding image-processing methods

The binary files of image-processing regarding pore-free recognition and Nups foci detection are available on http://www.riken.jp/brict/Takemoto/software/pore-free.html.

## Electronic supplementary material

Supplementary information
