# Supplementary material for: A statistical image analysis framework for pore-free islands derived from heterogeneity distribution of nuclear pore complexes
Source: Sci Rep. 2017 Nov 24;7:16315. doi: 10.1038/s41598-017-16386-2 (PMC5701208; doi:10.1038/s41598-017-16386-2)
Supplement: Supplementary file 1 — Supplementary information [file 41598_2017_16386_MOESM1_ESM.pdf]

Supplementary information

A statistical image analysis framework for pore-free islands derived from heterogeneity distribution of nuclear pore complexes

Yasuhiro Mimura[1*], Satoko Takemoto[2*], Taro Tachibana[3], Yutaka Ogawa[1], Masaomi Nishimura[2], Hideo Yokota[2**], Naoko Imamoto[1**]

[1]Cellular Dynamics Laboratory, RIKEN Advanced Science Institute, Saitama, Japan

[2]Image Processing Research Team, RIKEN Centre for Advanced Photonics, Saitama, Japan

[3]Department of Bioengineering, Graduate School of Engineering, Osaka City University, Osaka, Japan

* These authors contributed equally to this work

**Equal correspondence

Supplementary Figure 1

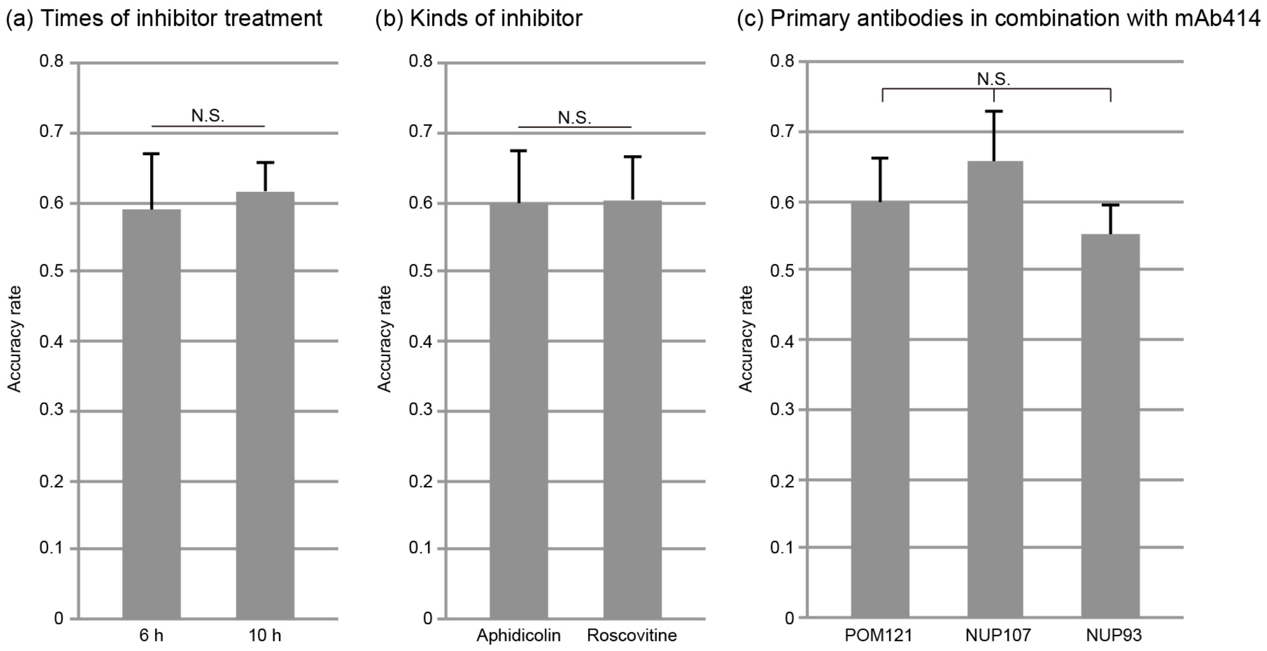

Fig. S1 Comparisons of averaged accuracy rates (related to Fig. 2).

Averaged accuracy rates were calculated for the indicated experimental conditions: (a) time of inhibitor treatment (n = 7 for 6 h and n = 6 for 10 h), (b) kind of inhibitor (n = 7 for aphidicolin and n = 6 for roscovitine), and (c) antibody combinations (n = 5 for POM121; n = 4 for NUP107; n = 4 for NUP93). Averaged accuracy rates calculated in (a) and (b) were analysed using a non-paired Student's T-test and that in (c) using one-way ANOVA; error bar, standard deviation; N.S., not significant ($P > 0.05$).

Supplementary Figure 2

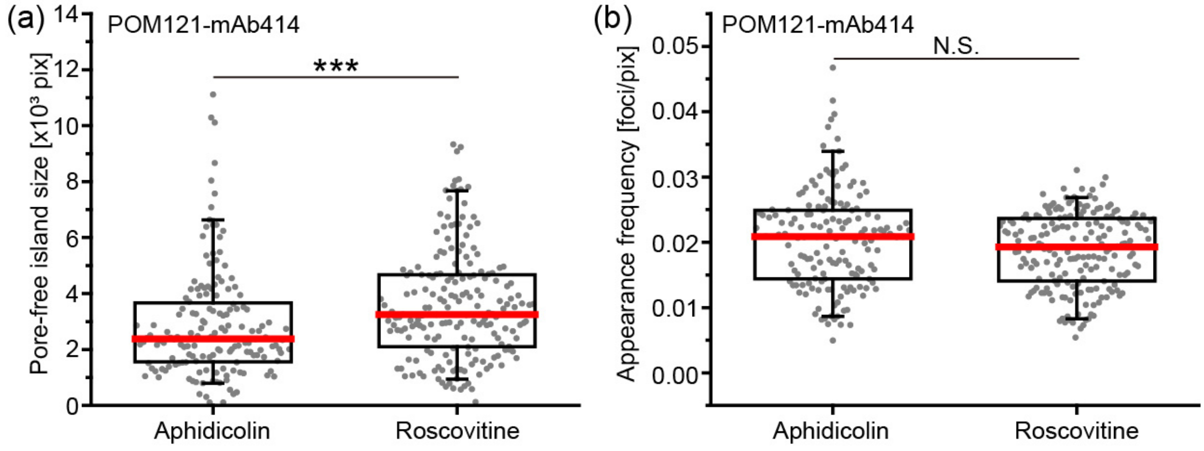

Fig. S2 Confirmation of the effects of secondary antibody swapping.

Cells treated with aphidicolin or roscovitine for 6 h were incubated with mAb414 and anti-POM121 antibodies. Then, the mAb414 antibody-recognised foci and anti-POM121 antibody-recognised foci were visualised using Alexa488- and Alexa594-conjugated secondary antibodies, respectively, and the size of pore-free islands and the appearance frequency of POM121 foci were measured. Bee-swarm and box-whisker plots for the size of pore-free islands (a) and appearance frequency of POM121-foci in pore-free islands (b). The box shows interquartile range (25% and 75%) of the data set and red bars show the median of the data set. Whiskers show 95% and 5% of the data set, respectively. Data were analysed using the Brunner-Munzel test; ***$P < 0.001$; N.S., not significant ($P > 0.05$).

Supplementary Figure 3

(a)

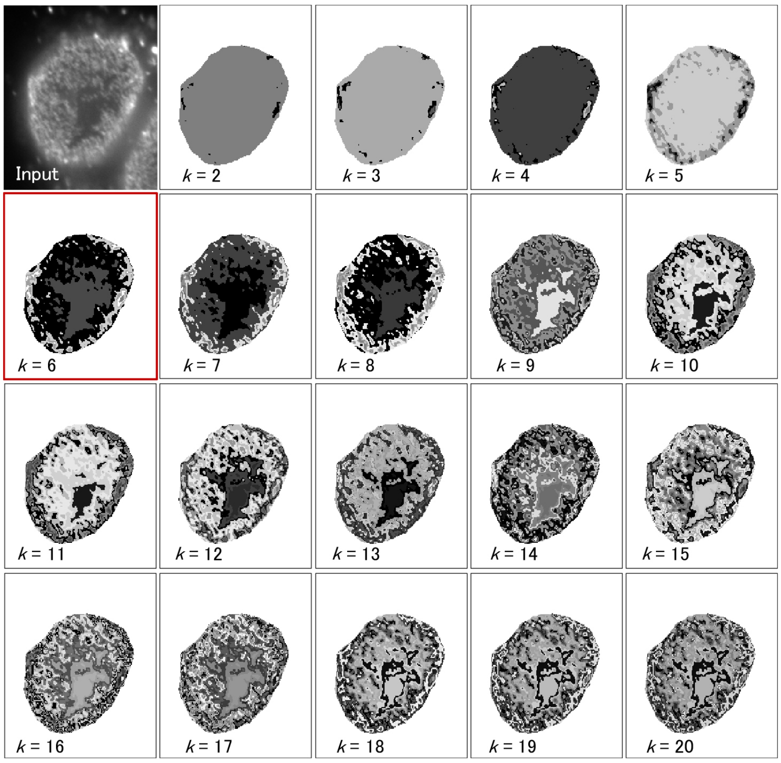

(b)

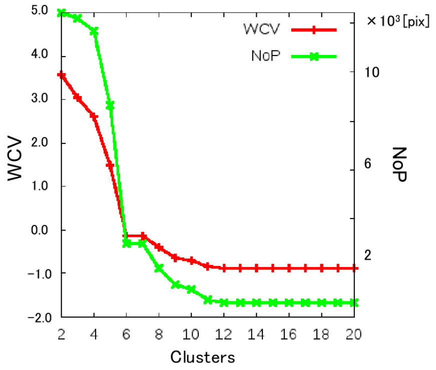

(c)

Num of clusters: k	WCV_k	Diffwcv : $WCV_{k+1} - WCV_k$	Num of Pix.
2	3.573	-0.517	12426
3	3.056	-0.445	12178
4	2.611	-1.122	11695
5	1.489	-1.633	8634
6	-0.144	0.001	2987
7	-0.143	-0.25	2989
8	-0.393	-0.24	1991
9	-0.633	-0.064	1338
10	-0.697	-0.144	1141
11	-0.841	-0.031	690
12	-0.872	0	592
13	-0.872	0	592
14	-0.872	-0.008	592
15	-0.88	0.008	568
16	-0.872	0	592
17	-0.872	-0.008	592
18	-0.88	0	568
19	-0.88	0	568
20	-0.88	N/A	568

(d)

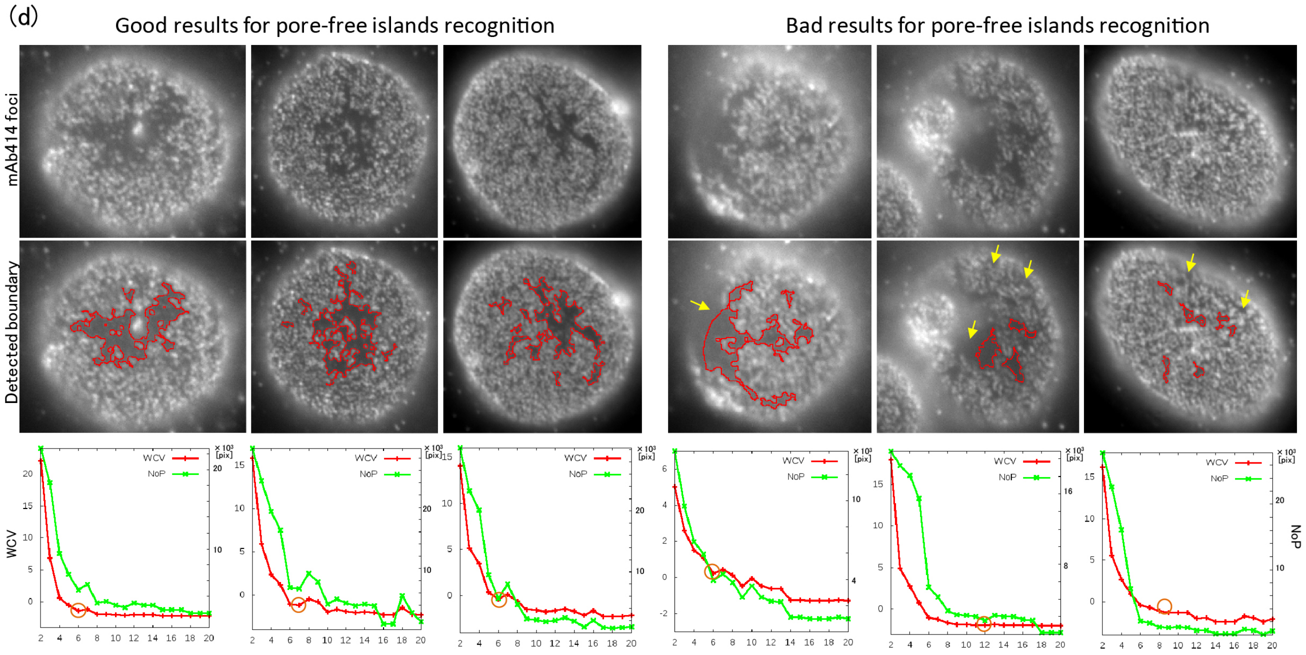

Fig. S3 Decision rule for the number of clusters in K-means for pore-free island recognition.

(a) Example of pore-free island recognition. The images show an input image and 19 types of clustering results with k = 2～20. Each result shows the segmented regions with the same number of grayscale intensities as k.

(b) Graph showing the line chart of the total within-class variation (WCV) and the number of pixels (NoP) inside the recognized pore-free islands at various numbers of k. WCV was calculated using linear combination of each normalized WCV, namely, z-score normalization for each within-class variation of image moment feature, mean, variance, and second order central image moment. In this case, per our proposed decision rule for the number of clusters, $k = 6$ was selected as the appropriate result for pore-free island recognition. Scale bar, 5 μm.

(c) Table showing the values of WCV shown in (b) and the difference WCV ($Diff_{wcv}$) between the subsequent clusters regarding k.

(d) Examples of good/bad recognition results. The first row shows the mAb414-staining images. The second row shows the recognition results of pore-free islands. The red lines indicate the boundaries of the recognized pore-free islands. The graphs shown in the third row are the line charts of WCV and NoP. In these graphs, the selected number of clusters for K-means are indicated using the orange dots circles.

Supplementary Figure 4

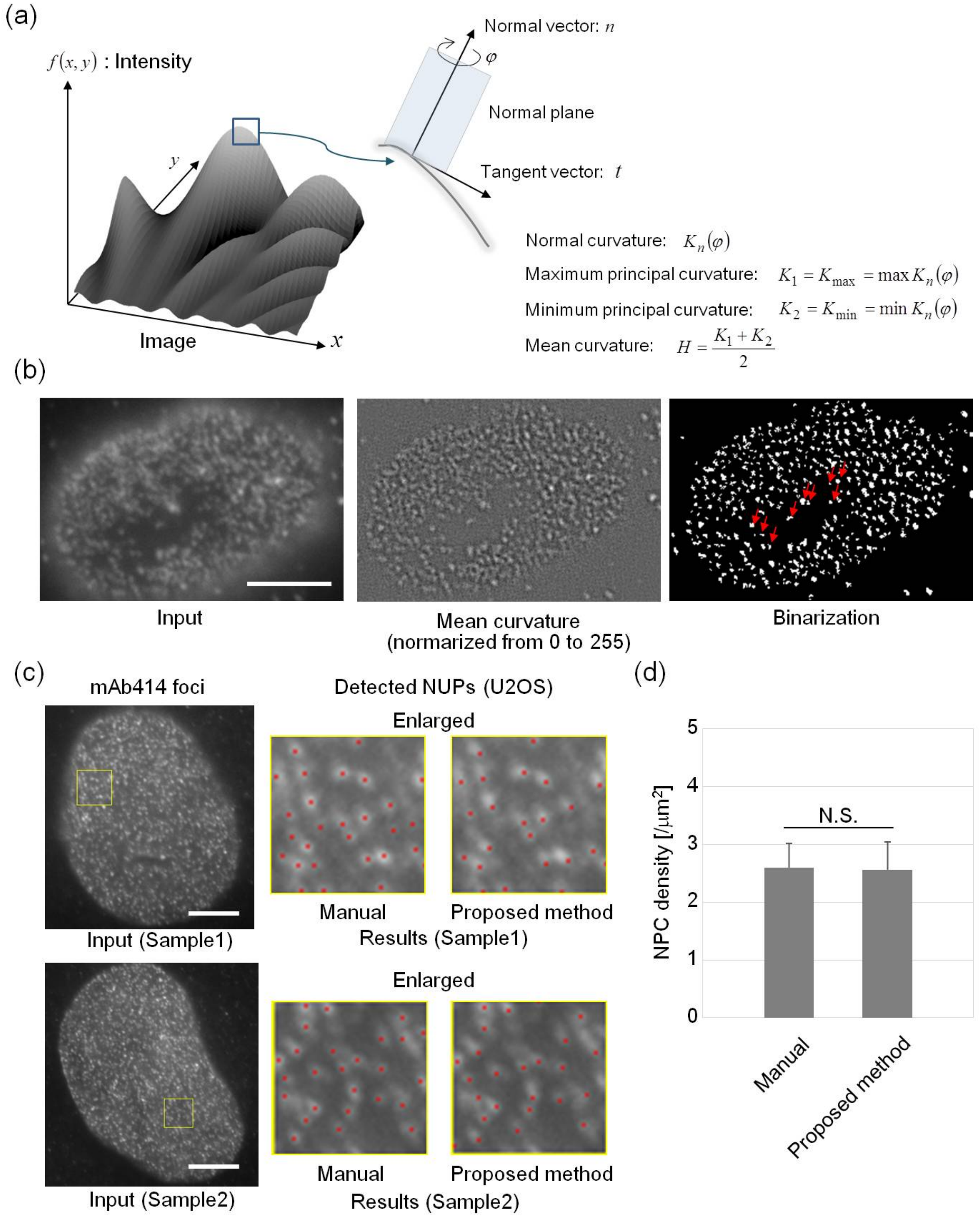

Fig. S4 Automatic Nups detection using the image features based on differential geometry.

(a) Conceptual diagram and its definition of curvature-based image features.

(b) Example of Nup-staining image (left) and its converted image, with pixels showing the mean curvature at the point enclosed by 5 × 5 pixels (centre). The mean curvature was normalised into eight bits. The right image shows the binarization result of the centre image obtained using the Otsu method. Arrows indicate the position of detected Nup foci. Scale bar, 5μm.

(c) Validation of the precision of our automatic Nups detection method using U2OS cells. U2OS cells were immunostained with mAb414 antibody to stain the NPCs (left input images). The rectangles in the input images indicate the position of the enlarged images represented on their right. The NPCs in the enlarged images were manually counted (Manual) or were counted using our proposed method (Proposed method), and the results are shown with red dots. Scale bar, 5μm.

(d) The calculated NPC densities from the manually counted result (Manual) or the result obtained using our proposed method (Proposed method) were plotted. The data were assessed using paired samples Student's T-test; N.S., not significant ($P > 0.05$); $n = 49$.

Supplementary Figure 5

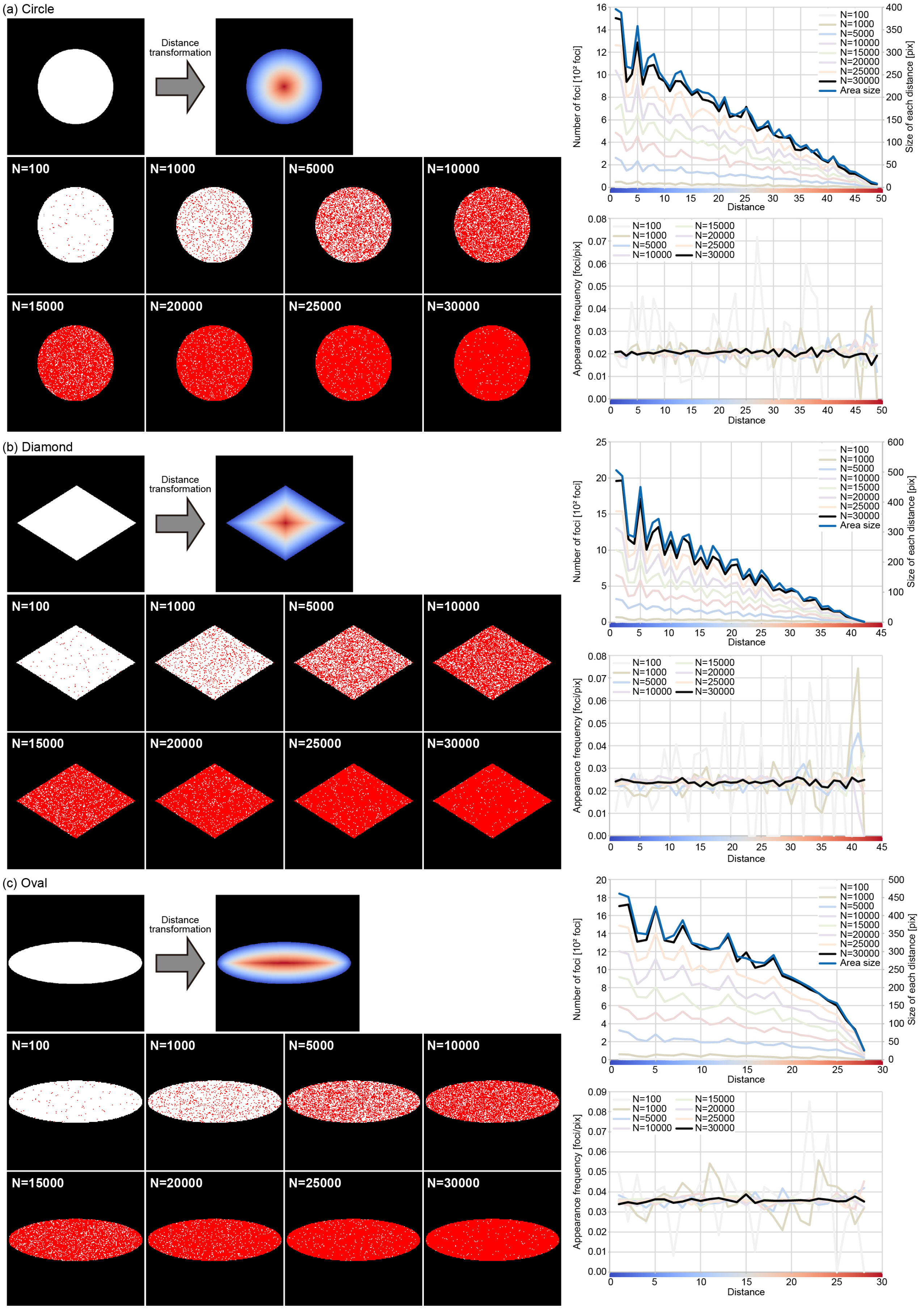

Fig. S5 Demonstration of the randomness of our foci placement program using artificial pore-free island.

Up to 30,000 foci were generated in three shapes of artificial pore-free islands to validate its randomness—circle (a), diamond (b), and oval (c). N indicates the number of foci. The images in the top row in each artificial pore-free island show schematic representation of the result after distance transformation. The images in the middle and bottom rows in each artificial pore-free island show the visual results generating foci up to the indicated number within each pore-free island. The top graphs in each artificial pore-free island were plotted against the number of foci and the size of each distance. The size of each distance and the number of foci in each distance were represented with blue and other colours, respectively. The bottom graphs in each artificial pore-free island were plotted against the appearance frequencies of generating foci in each distance.

TableS1 Summary of the statistical data of pore-free islands.

Sample name	Antibodies 1st	Antibodies 2nd	Inhibitor	Treatment time	No. of images	No. of adopted images	Accuracy rate	Median value of pore-free island [pix][1]	Median number of Nup foci in pore-free island	Median value of bottom surface [pix][1]	Appearance frequency of Nup foci [foci/pix]
POM121-mAb414 (ex1)	POM121	Alexa-488	Aphidicolin	6 h	80	43	0.54	2227	14	29041	0.00607
	mAb414	Alexa-594									
POM121-mAb414 (ex2)	POM121	Alexa-488	Aphidicolin	6 h	246	148	0.60	2441.5	16	32500	0.00683
	mAb414	Alexa-594									
POM121-mAb414	POM121	Alexa-488	Roscovitine	6 h	187	101	0.54	2902	13	33271	0.00509
	mAb414	Alexa-594									
POM121-mAb414	POM121	Alexa-594	Aphidicolin	6 h	268	156	0.58	2381.5	51.5	35097.5	0.02086
	mAb414	Alexa-488									
POM121-mAb414	POM121	Alexa-594	Roscovitine	6 h	270	189	0.70	3264	61	36593	0.01928
	mAb414	Alexa-488									
POM121-mAb414	POM121	Alexa-488	Aphidicolin	10 h	273	188	0.69	2363.5	17.5	34952	0.00822
	mAb414	Alexa-594									
POM121-mAb414	POM121	Alexa-488	Roscovitine	10 h	313	194	0.62	3471.5	17	33330.5	0.00576
	mAb414	Alexa-594									
NUP107-mAb414	NUP107	Alexa-488	Aphidicolin	6 h	221	154	0.70	2354	18	34820.5	0.00751
	mAb414	Alexa-594									
NUP107-mAb414	NUP107	Alexa-488	Roscovitine	6 h	223	157	0.70	3055	21	35589	0.00749
	mAb414	Alexa-594									
NUP107-mAb414	NUP107	Alexa-488	Aphidicolin	10 h	300	180	0.60	2430.5	17	34014.5	0.00808
	mAb414	Alexa-594									
NUP107-mAb414	NUP107	Alexa-488	Roscovitine	10 h	295	183	0.62	3602	21	34528	0.00598
	mAb414	Alexa-594									
NUP93-mAb414	NUP93	Alexa-488	Aphidicolin	6 h	275	138	0.50	1984	4	38363	0.00213
	mAb414	Alexa-594									
NUP93-mAb414	NUP93	Alexa-488	Roscovitine	6 h	377	202	0.54	3330.5	8	38378	0.00229
	mAb414	Alexa-594									
NUP93-mAb414	NUP93	Alexa-488	Aphidicolin	10 h	305	172	0.56	2629	8	46678	0.00368
	mAb414	Alexa-594									
NUP93-mAb414	NUP93	Alexa-488	Roscovitine	10 h	299	180	0.60	3347.5	9	42232.5	0.00234
	mAb414	Alexa-594									
mAb414-18h	-	-	-	-	211	138	0.65	1684.5	-	31407	-
	mAb414	Alexa594									

[1] Pixel size, 0.06469 × 0.06469 μm^2

TableS2 Statistical evaluation of the appearance frequency distribution of NPC intermediates between the observed and simulated datasets.

Antibody	Inhibitor	Treatment time	P value[1]	
			Proximal region	Distal region
NUP93 x mAb414	Aphidicolin	6 h	0.3517	0.02222
	Roscovitine	6 h	0.1678	0.1641
	Aphidicolin	10 h	0.3517	0.001632
	Roscovitine	10 h	0.1678	0.003323
NUP107 x mAb414	Aphidicolin	6 h	0.7301	0.03663
	Roscovitine	6 h	0.5361	0.002342
	Aphidicolin	10 h	0.4175	0.1641
	Roscovitine	10 h	0.5882	0.0337
POM121 x mAb414	Aphidicolin	6 h	0.1678	0.03663
	Roscovitine	6 h	0.3517	0.03663
	Aphidicolin	10 h	0.7869	0.4175
	Roscovitine	10 h	0.5882	0.002342

[1] P values were evaluated using Two-sample Kolmogorov-Smirnov test.
